# Impact of national malaria control scale-up programmes in Africa: magnitude and attribution of effects

**DOI:** 10.1186/1475-2875-9-299

**Published:** 2010-10-27

**Authors:** Richard W Steketee, Carlos C Campbell

**Affiliations:** 1 Malaria Control and Evaluation Partnership in Africa (MACEPA), PATH 2201 Westlake Avenue, Suite 200, Seattle, WA 98121 USA

## Abstract

**Background:**

Since 2005, malaria control scale-up has progressed in many African countries. Controlled studies of insecticide-treated mosquito nets (ITNs), indoor residual spraying (IRS), intermittent preventive treatment during pregnancy (IPTp) and malaria case management suggested that when incorporated into national programmes a dramatic health impact, likely more than a 20% decrease in all-cause childhood mortality, was possible. To assess the extent to which national malaria programmes are achieving impact the authors reviewed African country programme data available through 2009.

**Methods:**

National survey data, published literature, and organization or country reports produced during 2000-2009 were reviewed to assess available malaria financing, intervention delivery, household or target population coverage, and reported health benefits including infection, illness, severe anaemia, and death.

**Results:**

By the end of 2009, reports were available for ITN household ownership (n = 34) and IPTp use (n = 27) in malaria-endemic countries in Africa, with at least two estimates (pre-2005 and post-2005 intervals). Information linking IRS and case management coverage to impact were more limited. There was generally at least a three-fold increase in household ITN ownership across these countries between pre-2005 (median of 2.4% of households with at least one ITN) and post-2005 (median of 32.5% of households with at least one ITN). Ten countries had temporal data to assess programme impact, and all reported progress on at least one impact indicator (typically on mortality); in under-five year mortality rates most observed a decline of more than 20%. The causal relationship between malaria programme scale-up and reduced child illness and mortality rates is supported by biologic plausibility including mortality declines consistent with experience from intervention efficacy trials, consistency of findings across multiple countries and different epidemiologic settings, and temporal congruity where morbidity and mortality declines have been documented in the 18 to 36 months following intervention scale-up.

**Conclusions:**

Several factors potentially have contributed to recent health improvement in African countries, but there is substantial evidence that achieving high malaria control intervention coverage, especially with ITNs and targeted IRS, has been the leading contributor to reduced child mortality. The documented impact provides the evidence required to support a global commitment to the expansion and long-term investment in malaria control to sustain and increase the health impact that malaria control is producing in Africa.

## Background

**Figure 1:**
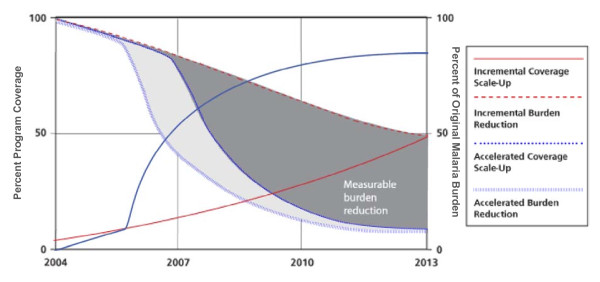
**Malaria programme scale-up: relationship between coverage and expected burden reduction**. *Note: Programme scale-up showing gradual incremental coverage increases (in red) versus rapid and accelerated coverage increases (in blue); the expected concomitant burden reduction suggests that the rapid and accelerated approach leads to an increased burden reduction and added benefit (in gray) from accelerated scale-up. This graphic assumes a direct relationship between population-based intervention coverage and programme impact; it also suggests a time lag between achieving high coverage and having the population experience the intervention benefit (perhaps across a malaria transmission season)*.

The Roll Back Malaria (RBM) Partnership endorsed "Scale Up for Impact" (SUFI) as the approach to rapidly increase access to and use of malaria control interventions (as referenced in its Global Malaria Action Plan [1]). SUFI is predicated on the rapid deployment of a package of proven malaria interventions to high levels of coverage to quickly achieve the optimal health effects based on evidence from controlled trials (see Figure 1). Programme impact accrues from multiple intervention effects and, when scaled to high coverage nationwide, is expected to have greater impact than any one intervention alone.

Experience in national programme scale-up has identified key determinants of achieving impact by assuring that policies and mechanisms are in place to deploy the full package of proven malaria interventions. The preventive interventions--insecticide-treated nets (ITNs) [2-5], indoor residual spraying (IRS) [6-9], and prevention in pregnancy with intermittent preventive treatment (IPTp) and ITNs [10-12]--all have documented efficacy from controlled trials. Malaria case management is clearly assumed to be effective as an intervention, but cannot be tested in placebo-controlled trials because not giving treatment to a malaria-infected person is unacceptable; as a consequence, efficacy estimates for this intervention are not based on clinical trials. Further, the only data available are either summarized expert opinion [13] or time-sequence data from health facilities to provide estimates of protective efficacy for case management with mortality as the outcome [14-17]. These efficacy estimates have been recently reviewed and calibrated for use in models that can estimate the expected benefit in lives saved based on intervention coverage achieved [18].

From 2005 through 2009, a number of countries have scaled up programme coverage, particularly with prevention interventions, to levels that have achieved measureable impact. Similarly, data were available from large-scale, but sub-national, malaria control scale-up efforts where impact was documented [19]. In analysing the results of these national and large sub-national programmes, it is important to critically assess the causal relationships across a range of national estimates and programming contexts. This report summarizes recent progress in sub-Saharan African countries and makes the case for attributing health impact to the malaria programme interventions.

## Methods

Information was reviewed from national household population-based surveys--including Demographic and Health Surveys (DHS) [20], Multiple Indicator Cluster Surveys (MICS) [21], Malaria Indicator Surveys (MIS) [22], and AIDS Indicator Surveys (AIS) [23]--as well as published literature and a variety of organization reports examining changes from 2000 through 2009 (with emphasis on the interval from 2004 through 2009) in reported delivery of malaria prevention and control products (ITNs, IRS, drugs for prevention and treatment), changes in intervention coverage rates, and reported health benefits (infection, illness, and death). Because malaria is largely a disease of the rural poor and because inequities in intervention delivery have historically resulted in rural and poor populations being underserved [24], the authors focused on estimates of intervention coverage in rural areas of the countries as reported in standard surveys. Standard outcome and impact measures have been set through an iterative review process managed by the RBM Monitoring and Evaluation Reference Group (MERG) [25-27]. The core health impact measures are: 1) all-cause mortality of children younger than five years (in Africa and similar high-endemic settings); 2) parasite prevalence within age groups (0-59 months or 6-59 months); 3) anaemia (Hb < 8.0 gm/dl) rates in young children (6-59 or 6-36 months of age); 4) outpatient case rates preferably with laboratory confirmation; 5) in-patient admission rates preferably with laboratory confirmation; and 6) case fatality rates preferably with laboratory confirmation of parasitaemia. Because rural populations are typically at highest risk of malaria and have historically benefited the least by disease control programme scale up, an analysis for rural areas is provided where it was available; "rural" was defined by each country using their national office of statistics' definitions for population-based surveys.

To assess the strength of the evidence for a causal link between the scale-up of malaria control interventions and reductions in child morbidity and mortality, considered established criteria for causal association were considered [28]. Several country experiences were reviewed in detail for Tanzania [29-33] and Zanzibar [34], Zambia [35-38], and Bioko Island (Equatorial Guinea) [39-41] to further address aspects of causal association.

## Results

### Malaria prevention programme scale-up in Africa

**Figure 2:**
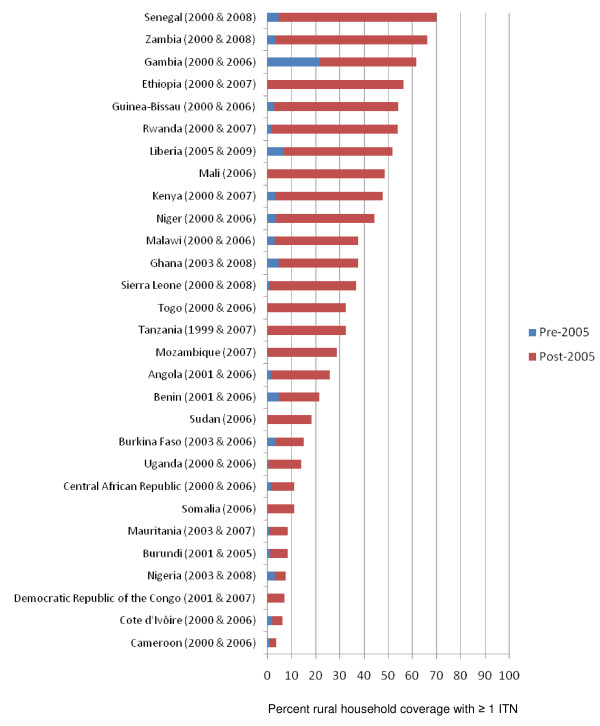
**Percent coverage in rural areas of countries where households own at least one insecticide-treated net (ITN): pre-2005 coverage level (blue) and post-2005 increase in coverage levels (red) from national survey data**. *Source: Demographic and Health Surveys (MACRO, http://www.measuredhs.com); Multiple Indicator Cluster Surveys (UNICEF, http://www.childinfo.org); and Malaria Indicator Surveys (RBM, http://www.rollbackmalaria.org)*.

**Figure 3:**
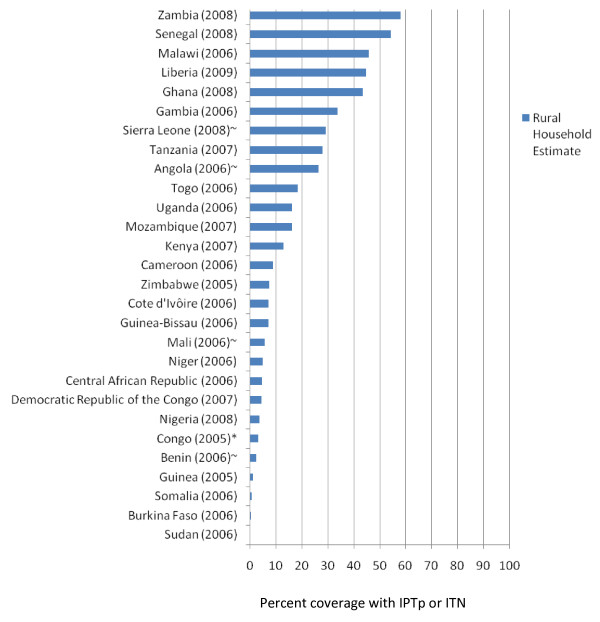
**Percent coverage in rural areas of the countries using prevention in pregnant women--either repeated use of intermittent preventive treatment during pregnancy (IPTp) or ITN use**. * These estimates were not specified as two or more doses of sulphadoxine-pyrimethamine received at antenatal clinic visit. ~These estimates reflect pregnant women sleeping under an ITN the night before the survey; all others are IPTp received among women giving birth in the past two years. *Source: Demographic and Health Surveys (MACRO, http://www.measuredhs.com), Multiple Indicator Cluster Surveys (UNICEF, http://www.childinfo.org), and Malaria Indicator Surveys (RBM, http://www.rollbackmalaria.org)*.

**Figure 4:**
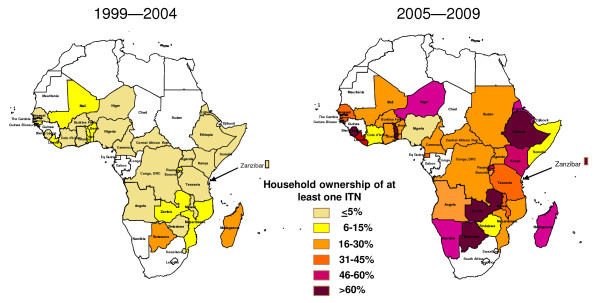
**Geographic distribution in Africa of household ownership of at least one insecticide-treated net (ITN) from national surveys in the intervals of 1999-2004 and 2005-2009**. *Source: Demographic and Health Surveys (MACRO, http://www.measuredhs.com), Multiple Indicator Cluster Surveys (UNICEF, http://www.childinfo.org), and Malaria Indicator Surveys (RBM, http://www.rollbackmalaria.org)*.

Many countries across sub-Saharan Africa have rapidly increased their malaria prevention coverage, particularly with ITNs, but also with IRS in targeted areas and with IPTp and ITNs in pregnant women. Figure 2 demonstrates the substantial progress since 2005 in scaling up ITN programmes; it shows national estimates for rural areas (where most of the malaria transmission occurs) in ITN household ownership in 33 countries in sub-Saharan Africa with 29 of these countries having at least two national estimates during the pre-2005 and 2005 through 2009 time interval. Prior to 2005, most countries recorded less than 5% of households having at least one ITN (median of 2.4% ownership). While only three countries exceeded 60% of households with at least one ITN in rural areas from 2005 through 2009, 10 exceeded 40% and 17 exceeded 25% of rural households with at least one ITN (median of 32.5% ownership for the post-2005 surveys). Figure 3 shows the progress in use of IPTp and/or ITNs in pregnant women in 28 countries; while nine countries have exceeded 25% use of IPTp or ITNs in rural pregnant women, 19 have coverage levels below 20%. Figure 4 shows ITN ownership estimates across the African continent by pre-2005 and 2005 through 2009 intervals to provide a profile of the geographic distribution of the scale-up. In general, this increase in coverage coincides with dramatic growth in external committed financing for malaria control from approximately US$100 million in 2003 to US$1.7 billion in 2009 [42]. The scale-up timing lags by approximately one year after the funding becomes available due to the time required for commodity procurement and distribution.

### Evidence for programme impact

Based both on country reports and those published in peer-reviewed journals [19], there is consistent temporal evidence for impact (e.g., morbidity and mortality reduction) as malaria programme coverage has increased in countries in the Africa region. Information from 2000-2008 on malaria control progress was reviewed from countries where data were available in the published literature. Some countries and sub-national regions (South Africa, Swaziland, southern Mozambique and Eritrea; see references [57-63] and [45,46] respectively) reported progress from 2000 through 2004. The majority of countries (Benin, Burundi [43,44], Ethiopia [47-50], Equitorial Guinea [39-41], Gambia [51,52], Kenya [53-56], Rwanda [64], Senegal [67], United Republic of Tanzania [29-34], and Zambia [35-38]) had demonstrable progress from 2005 through 2009. Four countries (Cameroon [68]; Congo [69]; Madagascar [70]; and Sudan [71]) had early reports of little or no progress and three countries (Burkina Faso [72], Nigeria [73] and Zimbabwe [74]) had early reports of worsening malaria; more recent information on improved funding and scale-up for malaria control has been reported from these countries but is not yet adequately documented.

**Figure 5:**
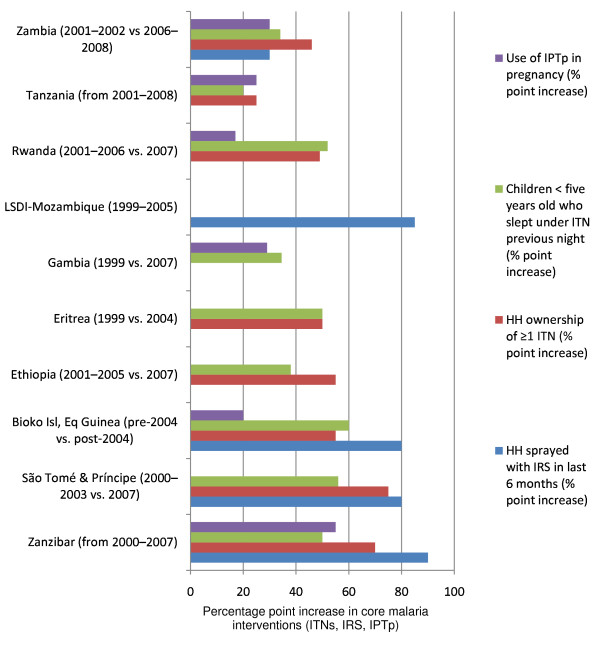
**Percentage point increases in core malaria interventions in 10 countries reporting substantial improvement with malaria intervention scale-up**. *Source: Demographic and Health Surveys (MACRO, http://www.measuredhs.com), Multiple Indicator Cluster Surveys (UNICEF, http://www.childinfo.org), Malaria Indicator Surveys (RBM, http://www.rollbackmalaria.org), and country reports*.

**Figure 6:**
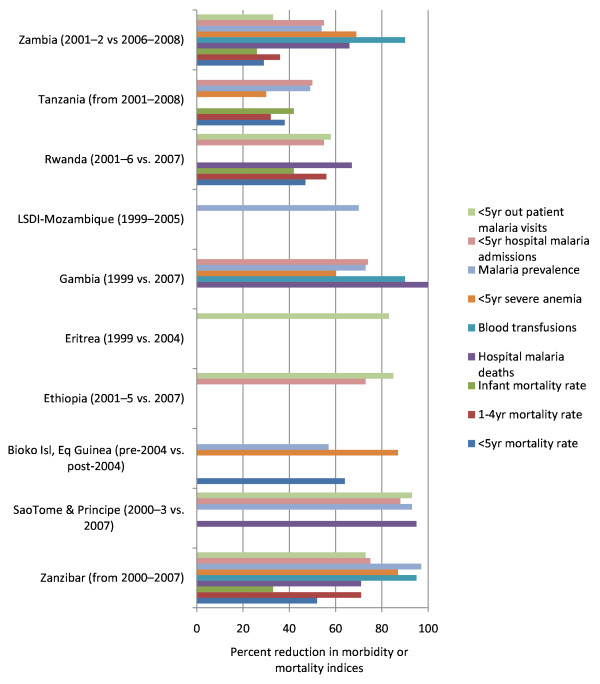
**Percent changes in key malaria indicators in countries with substantial malaria control programme scale-up**. *Source: Impact of national malaria control scale-up programs in Africa: magnitude and attribution of effects. Report for the Malaria Control and Evaluation Partnership in Africa (MACEPA)/PATH, Seattle, USA citing the following articles: Zambia *[38]*, Zanzibar *[34]*, Rwanda and Ethiopia *[48].

Countries and areas with published data on national intervention scale-up and impact include: Equatorial Guinea-Bioko Island, Eritrea, Ethiopia, Gambia, Kenya, Rwanda, São Tomé and Príncipe, South Africa-Swaziland-Mozambique in the Lubombo Spatial Development Initiative (LSDI) Project, Tanzania (mainland and Zanzibar), and Zambia. Figure 5 shows percentage point increases in coverage for malaria prevention interventions during the past decade for these 10 settings. Marked increases in IRS coverage were documented in the LSDI area and on the islands of Bioko, São Tomé and Príncipe, and Zanzibar; most countries relied predominately on ITN scale-up with some use of IRS and IPTp. Figure 6 shows the temporal reduction in childhood morbidity and mortality.

### Country vignette: Mainland Tanzania and Zanzibar

Malaria control scale-up has progressed on both mainland Tanzania [29-33] and on Zanzibar [34]. Mainland Tanzania with its large geographic area and population has yet to achieve full scale-up across all districts, but has increased household ownership of ITNs in both urban and rural areas. In 1999, the Tanzania DHS reported that while 20% of children under five years of age reportedly slept under a mosquito net, only 10% of these nets were treated with insecticide--thus there was an estimated 2% use of ITNs in 1999. By the 2007-2008 DHS, 39% (59% in urban and 33% in rural areas) of houses owned at least one ITN; and the use of IPTp in pregnant women had risen to 30% nationwide. Between 2006 and 2008, malaria parasite prevalence in children under five years of age declined by nearly 30% from 20% to 14%; and in some areas with higher household ITN ownership, the decline was more dramatic [33].

In contrast to the gradual improvement on mainland Tanzania, rapid deployment and high coverage of IRS and ITNs was achieved on Zanzibar, as has the use of diagnostics and artemisinin-based combination therapy (ACT). Zanzibar's dramatic scale-up of IRS and of ACT began in the 2003 through 2004 time interval and increasing household ownership of ITNs occurred between 2005 and 2006; increasing use of diagnostics began in the 2006 through 2007 interval. This scale-up coincided with documented reductions in child morbidity and mortality; this includes a 68% reduction in outpatient malaria diagnoses, a 75% reduction in malaria hospital admission, a 63% reduction in blood transfusion, and a 72% reduction in overall malaria-attributed mortality [34].

### Country vignette: Zambia

**Figure 7:**
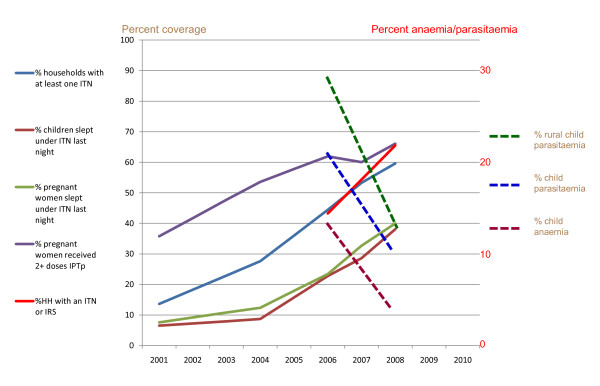
**Malaria intervention coverage rates from national surveys (2001-2008), and parasitaemia and anaemia rates from Malaria Indicator Surveys (2006 and 2008) in Zambia**. *Note: National survey data is available for 2001-2002, 2004, 2006, 2007, and 2008; interim annual estimates are linear extrapolations between known data points. HH: households; IPTp: intermittent preventive treatment in pregnancy; IRS: indoor residual spraying; ITN: insecticide-treated net; anaemia = Hb <8 gm/dl*.

From 2004 through 2008, Zambia increased household ITN ownership and use and IPTp coverage nation-wide; IRS was initially targeted to urban and peri-urban areas of 12 districts, eventually expanding to 36 of the 72 districts. ACT was initiated in 2004, but coverage of ACT for clinical or confirmed malaria cases has not experienced the same dramatic increase as that seen as a result of scale-up of prevention intervention coverage. Figure 7 shows this scale-up of coverage from 2001 through 2008: households owning at least one ITN (40% increase), households owning two or more ITNs (64% increase), households with IRS in the last 12 months (58% increase), and children and pregnant women sleeping under an ITN (80% and 85% increase, respectively).

**Figure 8:**
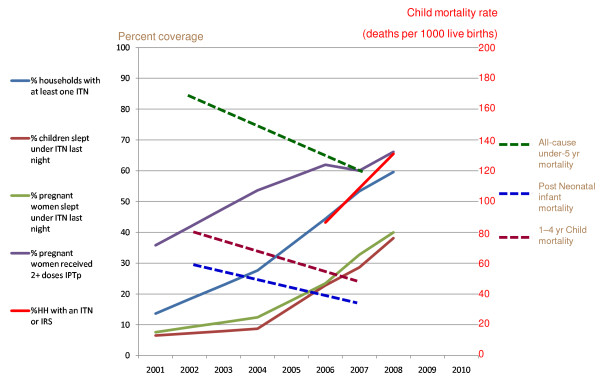
**Malaria intervention coverage rates per national surveys (2001-2008) and infant and child mortality rates (DHS 2001-2002 and 2007) in Zambia**. *Note: National survey data is available for 2001-2002, 2004, 2006, 2007, and 2008; interim annual estimates are linear extrapolations between known data points. IPTp: intermittent preventive treatment in pregnancy; IRS: indoor residual spraying; ITN: insecticide-treated net*.

The increased coverage rate of malaria prevention interventions from 2001 through 2008 coincided with reductions in child parasitaemia rates, which fell 53%, with most of the decline in rural areas, and child anaemia rates, which fell 68%, (see Figure 8). All-cause child mortality rates decreased between the DHS of 2001-02 and 2007 (reductions for 0-5 year age group, 1-11 month age group, and 1-4 year age group were 29%, 38%, and 36%, respectively). Specific analysis of the 2006 MIS data comparing households with ITNs versus households without ITNs showed that after adjusting for other known associations (including child age, urban-rural status, socio-economic status, and use of IRS), children living in houses with ITNs had significant reductions in morbidity in the two year interval: 28% fewer fever episodes in the previous two weeks, 39% lower rates of *P. falciparum *infection, and 28% less severe anaemia (Hb < 8 gm/dl). Households owning two or more ITNs had the best results in morbidity reduction. Reported paediatric hospital admissions, hospital deaths attributed to malaria (not all parasitologically confirmed), and paediatric outpatient visits for suspected malaria have similarly decreased, particularly in the 2006 through 2008 interval [37].

**Figure 9:**
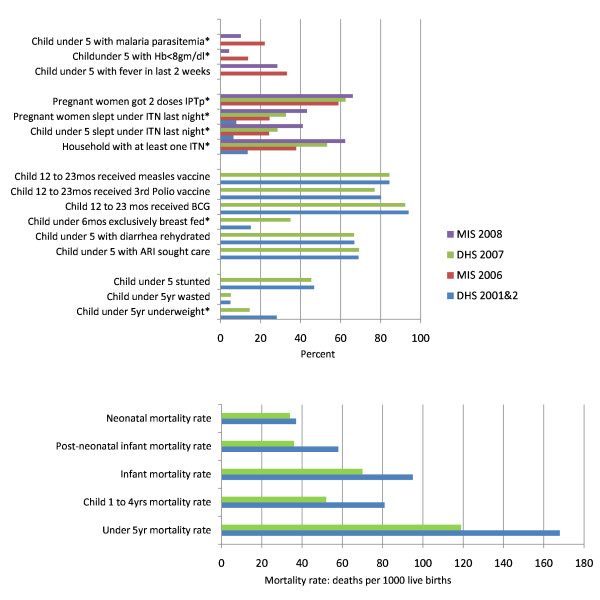
**Changes in intervention coverage, malaria morbidity and mortality, and other child health intervention coverage in Zambia between 2001 and 2008**. *Source: Demographic and Health Surveys (DHS) in 2001-2 and 2007; Malaria Indicator Surveys (MIS) in 2006 and 2008*.

Potential contributing factors to the decline in all-cause infant and child mortality were examined (Figure 9). Most health indicators reflected no or only modest improvement during the 2001 through 2008 interval; there were no changes in immunization coverage rates or treatment coverage rates for diarrheal or respiratory diseases during this interval. DHS and MIS survey data comparisons identified a nearly 50% reduction in the frequency of underweight children (from 28% to 15%) and a substantial increase in the rate of exclusive breastfeeding in infants less than six months of age (from 15% to 35%) from 2005 through 2007. While better breastfeeding practices may have contributed to improved child survival, this alone would not be expected to account for the observed large reductions in child mortality. The improvement in underweight children less than five years of age may have been due to a variety of factors including reduced malaria infections in the population (there were no changes in stunting and wasting). The largest change in intervention coverage that could have positively impacted child survival during this time interval and at this magnitude was the scale-up of malaria control interventions.

### Country vignette: Bioko Island, Equatorial Guinea

**Figure 10:**
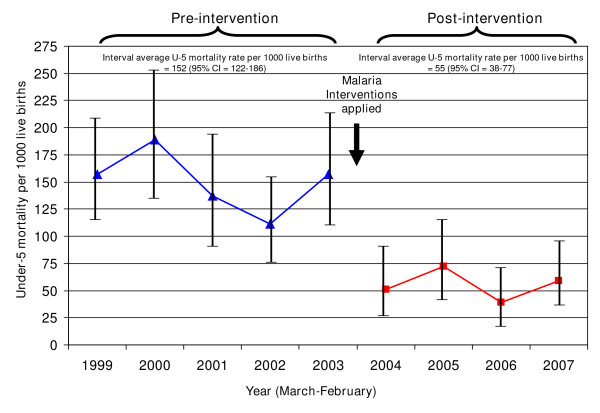
**Rates of all-cause under-five mortality on Bioko Island, Equatorial Guinea: pre- and post-malaria control interventions (IRS, ITNs, case management)**. *Source: Data taken directly from Table four in Kleinschmidt et al *[41]*. Malaria interventions included indoor residual spraying (IRS) and malaria case management with diagnosis and artemisinin-based combination therapy; increased household ownership of insecticide-treated mosquito nets (ITNs) was added in 2007*.

A substantial effort in Bioko Island started in 2004 with rapid, large-scale increases in twice-yearly IRS coverage, introduction of diagnostics, ACT, and use of IPTp; this was followed with ITN introduction in 2007. Figure 10 shows the immediate and steep reduction in all-cause child mortality (a 69% reduction, from a pre-intervention interval average of 152 to a post-intervention interval average of 55 per 1000 live births) associated with this malaria control scale-up. There were concomitant reductions in vector populations (over 90% reduced), mosquito sporozoite prevalence rates (over 90% reduced), parasite prevalence rates in children under five years of age (68% reduced), fever rates (89% reduced), and severe anaemia rates (Hb <8 gm/dl; 87% reduced) [41].

## Discussion

The past five years have witnessed the adoption of SUFI by many malaria-endemic African countries, deploying efficacious interventions and collecting comparable indicators that permit assessment of trends in child survival and malaria morbidity. There is remarkable consistency in impact across a diverse range of countries. Among the settings describing marked progress, three are islands (Bioko, São Tomé and Príncipe, and Zanzibar with a combined population of approximately 1.5 million), there is a three-country partnership (LSDI working with some districts in each of Mozambique, South Africa, and Swaziland); three are small countries (Eritrea, Gambia, and Rwanda with a combined population of approximately 16 million), and three are larger countries (Ethiopia, Tanzania, and Zambia with a combined population of more than 100 million). The epidemiologic and demographic breadth of these settings cannot yet be claimed to be representative of the diversity of malaria in Africa. But additional countries are progressing with malaria programme scale-up, and while it is understood that there is an inherent interval between programme scale-up and documenting programme outcomes, it is clear that a broad and representative profile of programme impact is emerging.

### Attribution of malaria control to the documented improvement in child survival

With the increasing documentation of geographic expansion of malaria programme scale-up in the Africa region, it is important to assess whether the observed health benefits are simply associations in time and place, or if convincing evidence exists to assert a causal relationship between the malaria control interventions and the dramatic documented health improvements.

Based on B.A. Hill's 1965 epidemiologic framework for causal inference [28], eight criteria need to be considered: 1) experimental evidence; 2) plausibility; 3) strength of association; 4) specificity of the association; 5) temporal congruity [or lack of temporal ambiguity]; 6) biologic gradient; 7) consistency of findings; and 8) coherence of the evidence. For the malaria control interventions, the first two criteria are addressed by the existing scientific studies that document the efficacy of interventions in controlled trials--that is, the interventions have been proven to reduce morbidity and mortality and it is fully plausible that their use in national settings can achieve the comparable results. The country data on intervention coverage scale-up and impact measures largely address criteria 3 through 8. For the strength and specificity of association and the temporal congruity (criteria 3, 4 and 5), a programme assessment may lack the most stringent criteria, however in the examples of Bioko Island, Zambia and Zanzibar, the association and time link between malaria control scale up and reductions in malaria morbidity and mortality is strong; and efforts to examine alternative explanations did not demonstrate substantial weaknesses with the associations. The timing of the intervention application and the benefit achieved is closely sequenced. The Bioko Island experience shows a dramatic and almost immediate drop in child mortality following the application of the malaria prevention and treatment package [41]. Similarly, the experience in Zanzibar showed a dramatic response to a combination of IRS, ITNs, and aggressive diagnosis and treatment [34]. Regarding biologic-gradient or dose-response, the sum of the multi-country information may not yet fully establish the dose-response effect where higher coverage is directly linked to higher impact. However, it is quite clear that low coverage of malaria interventions remains linked to limited improvements in child survival.

As for coherence of the evidence, it is critical to examine alternative explanations. Factors that could offer alternative explanations for the suggested link between malaria control scale-up and malaria morbidity and mortality reductions might include: 1) variations in rainfall and temperature; 2) broad socio-economic change; 3) changing HIV conditions; 4) other child health interventions discussed previously that might account for the differences; and/or 5) biologic changes in the malaria-vector-human cycle that is making malaria infection and illness less virulent. Many of the studies address and account for rainfall and temperature patterns and demonstrate that these are not plausible explanations of marked reductions in malaria during this time interval in most African countries. In Ethiopia, weather patterns are thought to have contributed to a substantial malaria epidemic from 2003 through 2005, so some of the findings there may be accounted for by this earlier period with high malaria as a comparison time for more recent scale-up and impact; however, this is not the case for other country settings. Some socio-economic change certainly occurred in Bioko Island with the growth of the oil industry, and improved copper prices in the 2005 through 2008 interval may have contributed indirectly in Zambia--but again, such socio-economic improvements occur in many countries but are not likely to explain the dramatic reduction in childhood mortality documented in the setting of SUFI. HIV rates have not dropped consistently across these countries, but improved treatment with anti-retroviral drugs may have contributed partially to the improved child survival and reductions in fever and malaria incidence and prevalence.

There are numerous challenges in a multi-country review of programme scale-up and its consequences. Largely, the study relied on national population-based survey data, which typically come from DHS, MICS and MIS, which have been repeatedly compared and updated to assure common wording and sequence of questions and standard reporting procedures; thus it is not expected that these different surveys introduced substantial bias in comparisons. For some questions, the timing of the survey is important. For example ITN use may vary substantially between the high transmission season and the hotter and dryer seasons when few or no mosquitoes prompt people to use their ITNs less; this would generally bias any results to being underestimates of actual ITN use in the peak transmission season. For counting malaria cases, however, a substantial variability is expected between and within countries as the introduction and expansion of diagnostics has increasingly excluded non-malarial fevers over time; unfortunately this leads to over-reporting of progress in reducing malaria cases and it is not possible to fully account for this in this summary review. In addition, child survival may well have been improving in some of these countries prior to intervention scale-up. The health systems must be reasonably robust to deliver the spectrum of malaria interventions through child health services, maternal health services, and community outreach and campaign distributions. These systems likely have been delivering other services (e.g., immunizations, vitamin A, and treatment of other illnesses) that improve child survival. While a clear majority alternative explanation for the improved health in these countries was not observed, each of these (and possibly additional factors) should be considered carefully by programmes examining the benefit of their malaria control scale-up.

Some of the observed measures of impact are at higher programme effectiveness levels than were predicted based on the coverage levels achieved and the known intervention efficacy data. This should not be surprising for several reasons. First, programmes are typically using multiple interventions simultaneously (ITNs, IRS, IPTp, and case management), not just one intervention that might have been tested in a controlled trial. Second, the controlled trials likely improved the services for both the intervention and comparison groups, thus making the efficacy estimate a conservative one. In contrast, programmes are being compared historically to times when much less was being done overall for malaria and possibly for other child health interventions, thus their observed gains appear large.

No single intervention can be credited with these dramatic improvements in the many countries; IRS may be credited in one country, ITNs in another, and effective drugs for case management in a third country. In fact, it is more likely that the composite package of preventive interventions (ITNs, IRS, IPTp) and treatment interventions (changing to highly effective drugs and using quality diagnostics) is responsible for the level of mortality reduction in individual countries. In some settings the benefit has been attributed to a specific intervention but this may be because countries have not clearly accounted for the role of the other interventions. For example, in most countries with marked improvement of case management, it is likely that the use of good diagnostics is responsible for much of the decline in reported malaria cases - by leading to the exclusion of non-malaria fevers from that case count. While scale-up of case management may have been the weakest of the efforts to date, recent emphasis on diagnostics may help further address this in the coming years. Of note, the overall effect of malaria control has been generated largely through the reduction of malaria transmission--both vector control and aggressive diagnosis and treatment in places like Zanzibar have contributed substantially to reduced transmission. It is inevitable that programme orientation to sustain the current gains will require an intense focus on transmission reduction.

## Conclusion

Coincident with the dramatic increase in funding over the past five years, African governments and their partners have documented the health impact that malaria control can produce by a coordinated national scale-up of the package of malaria control interventions. The important observation is that the evolving malaria control approach, SUFI, is robust in terms of predictably producing health impact and this can be led by national governments with partners. It can be confidently stated that national malaria programme scale-up has achieved substantial impact across a growing array of African countries. The reductions in child mortality are consistent with or even greater than the estimated 20% reduction in all-cause child mortality predicted from the controlled trials of ITNs. In 2010, hundreds of thousands of African children will not die of malaria because of recent national investments. This represents an impressive health impact across the region achieved through national-scale implementation of effective prevention and treatment measures. Malaria control with the current array of interventions represents a compelling investment that predictably prevents childhood deaths. But malaria control currently requires daily attention, the ITN must be hung tomorrow just as it was last night, and it must be replaced when it loses efficacy. The global malaria community now understands that malaria control is indeed the best buy currently in terms of child survival in Africa; and much still remains to be done in many countries including countries with large populations and persistent high malaria burdens. Malaria control cannot be sustained as an open-ended recurrent cost for donors and governments. The critical test will be developing the country experience with programming for incremental elimination of transmission to sustain the benefits of malaria control for future generations of African children.

## List of abbreviations

Abbreviations used in this report include: ACT: Artemisinin-based combination therapy; AIS: AIDS indicator survey; DHS: Demographic and Health Survey; Hb: Hemoglobin; HIV: Human immunodeficiency virus; IPTp: Intermittent preventive treatment during pregnancy; IRS: Indoor residual spraying; ITN: Insecticide-treated mosquito net; LSDI: Lubombo Spatial Development Imitative; MERG: RBM Monitoring and Evaluation Reference Group; MICS: Multiple indicator cluster survey; MIS: Malaria indicator survey; RBM: Roll Back Malaria; SUFI: Scale up for impact.

## Conflict of interests

The authors declare that they have no competing interests.

## Authors' contributions

RWS and CCC both contributed to overall design, assembly of information, writing of the report and reviewing of the final manuscript.

## References

[B1] Roll Back Malaria Partnership Global Malaria Action Plan. Geneva 2008

[B2] Lengeler C Insecticide-treated bed nets for preventing malaria The Cochrane Library 2007 3 10.1002/14651858.CD000363.pub2 15106149

[B3] Hawley WA Phillips-Howard PA ter Kuile F Terlouw DJ Vulule JM Ombok M Nahlen BL Gimnig JE Kariuki SK Kolczak MS Hightower AW Community-wide effects of permethrin-treated bednets on child mortality and malaria morbidity in Western Kenya Am J Trop Med Hyg 2003 68 121 127 12749495

[B4] Killeen GF Smith TA Ferguson HM Mshinda H Abdulla S Lengeler C Kachur SP Preventing childhood malaria in Africa by protecting adults from mosquitoes with insecticide-treated nets PLoS Med 2007 4 e229 10.1371/journal.pmed.0040229 17608562 PMC1904465

[B5] ter Kuile FO Terlouw DJ Phillips-Howard PA Hawley WA Friedman JF Kolczak MS Kariuki SK Shi YP Kwena AM Vulule JM Nahlen BL Impact of permethrin-treated bed nets on malaria and all-cause morbidity in young children in an area of intense perennial malaria transmission in western Kenya: cross-sectional survey Am J Trop Med Hyg 2003 68 100 107 12749492

[B6] Lengeler C Sharp B Indoor residual spraying and insecticide-treated nets Technical Report: Reducing Malaria's Burden: evidence of effectiveness for decision makers 2003 Washington, DC: Global Health Council http://www.globalhealth.org/assets/publications/malaria.pdf (accessed June 28, 2010)

[B7] Kolaczinski K Kolaczinski J Kilian A Meek S Extension of indoor residual spraying for malaria control into high transmission settings in Africa Trans R Soc Trop Med Hyg 2007 101 852 853 10.1016/j.trstmh.2007.04.003 17507065

[B8] Fontaine RE Pull JH Payne D Pradhan GD Joshi GP Pearson JA Thymakis MK Camacho ME Evaluation of fenitrothion for the control of malaria Bull World Health Organ 1978 56 445 452 308409 PMC2395585

[B9] Maharaj R Mthembu DJ Sharp BL Impact of DDT re-introduction on malaria transmission in KwaZulu-Natal S Afr Med J 2005 95 871 874 16344885

[B10] Garner P Gülmezoglu AM Drugs for preventing malaria-related illness in pregnant women and death in the newborn Cochrane Database Syst Rev 2003 1 CD000169 Update in *Cochrane Database Syst Rev *2006, **4**:CD000169 12535391 10.1002/14651858.CD000169

[B11] ter Kuile FO Terlouw DJ Phillips-Howard PA Hawley WA Friedman JF Kolczak MS Kariuki SK Shi YP Kwena AM Vulule JM Nahlen BL Impact of permethrin-treated bed nets on malaria and all-cause morbidity in young children in an area of intense perennial malaria transmission in western Kenya: cross-sectional survey Am J Trop Med Hyg 2003 68 100 107 12749492

[B12] Gamble C Ekwaru PJ Garner P ter Kuile FO Insecticide-treated nets for the prevention of malaria in pregnancy: a systematic review of randomised controlled trials PLoS Med 2007 4 e107 10.1371/journal.pmed.0040107 17388668 PMC1831739

[B13] Sudre P Breman JG Koplan JP Delphi survey of malaria mortality and drug resistance in Africa Lancet 1990 335 722 10.1016/0140-6736(90)90833-Q 1969074

[B14] Zucker JR Lackritz EM Ruebush TK II Hightower AW Adungosi JE Were JBO Metchock B Patrick E Campbell CC Childhood mortality during and after hospitalization in Western Kenya: effect of malaria treatment regimens Am J Trop Med Hyg 1996 55 655 660 9025694 10.4269/ajtmh.1996.55.655

[B15] Kidane G Morrow RH Teaching mothers to provide home treatment of malaria in Tigray, Ethiopia: a randomised trial Lancet 2000 356 550 555 10.1016/S0140-6736(00)02580-0 10950232

[B16] Price RN Nosten F Luxemburger C ter Kuile FO Paiphun L Chongsuphajaisiddhi T White NJ Effects of artemisinin derivatives on malaria transmissibility Lancet 1996 347 1654 1658 10.1016/S0140-6736(96)91488-9 8642959

[B17] Cairns M Carneiro I Milligan P Owusu-Agyei S Awine T Gosling R Greenwood B Chandramohan D Duration of protection against malaria and anaemia provided by intermittent preventive treatment in infants in Navrongo, Ghana PloS One 2008 3 e2227 10.1371/journal.pone.0002227 18493597 PMC2375060

[B18] Eisele TP Larsen D Steketee RW Protective efficacy of interventions for preventing malaria mortality in children in *Plasmodium falciparum *endemic areas Int J Epidemiol 2010 39 Suppl 1 i88 101 10.1093/ije/dyq026 20348132 PMC2845865

[B19] O'Meara WP Mangeni JN Steketee RW Greenwood B Changes in the burden of malaria in sub-Saharan Africa Lancet Infect Dis 2010 10 545 55 10.1016/S1473-3099(10)70096-7 20637696

[B20] IFC MACRO, Demographic and Health Surveys http://www.measuredhs.com/(accessed June 28, 2010)

[B21] UNICEF, Multiple Indicator Cluster Surveys http://www.childinfo.org/mics.html (accessed June 28, 2010)

[B22] RBM, Malaria Indicator Surveys http://rbm.who.int/toolbox/toolbox_MonitoringAndEvaluation.html?keyarea=Monit oring and Evaluation Survey Tools (accessed June 28, 2010)

[B23] IFC MACRO, AIDS Indicator Surveys http://www.measuredhs.com/(accessed June 28, 2010)

[B24] Steketee RW Eisele TP Is the scale up of malaria intervention coverage also achieving equity? PLoS One 2009 4 e8409 10.1371/journal.pone.0008409 20027289 PMC2791860

[B25] Roll Back Malaria Partnership Framework for monitoring progress and evaluating outcomes and impact. Geneva 2000

[B26] Roll Back Malaria Partnership The Abuja declaration and the plan of action From The African summit on Roll Back Malaria, Abuja, 25 April 2000 2000 17

[B27] Roll Back Malaria Partnership Guidelines for core population coverage indicators for Roll Back Malaria: to be obtained from household surveys 2006 Calverton, Maryland: Roll Back Malaria, Measure Evaluation, World Health Organization and UNICEF

[B28] Hill AB The environment and disease: Association or causation? Proc R Soc Med 1965 58 295 300 14283879 10.1177/003591576505800503 PMC1898525

[B29] Alilio MS Kitua A Njunwa K Medina M Rønn AM Mhina J Msuya F Mahundi J Depinay JM Whyte S Krasnik A Bygbjerg IC Malaria control at the district level in Africa: the case of the Muheza district in northeastern Tanzania Am J Trop Med Hyg 2004 71 Suppl 2 205 213 15331839

[B30] Lusingu JP Vestergaard L Mmbando BP Gesase S Ishengoma D Drakeley CJ A declining burden of malaria in northeastern Tanzania57th Annual Meeting of the American Society of Tropical Medicine and Hygine, New Orleans 2008 Abstract 601

[B31] Maegga BT Cox J Malley KD Malaria in the southern highlands of Tanzania: a review of hospital records Tanzan Health Res Bull 2005 7 125 132 16941937 10.4314/thrb.v7i3.14249

[B32] Killeen GF Tami A Kihonda J Okumu FO Kotas ME Grundmann H Kasigudi N Ngonyani H Mayagaya V Nathan R Abdulla S Charlwood JD Smith TA Lengeler C Cost-sharing strategies combining targeted public subsidies with private-sector delivery achieve high bednet coverage and reduced malaria transmission in Kilombero Valley, southern Tanzania BMC Infect Dis 2007 7 e121 10.1186/1471-2334-7-121 PMC2211306 17961211

[B33] Smithson P Down but not out. The impact of malaria control in Tanzania Ifakara Health Institute Spotlight 2009 2

[B34] Bhattarai A Ali AS Kachur SP Mårtensson A Abbas AK Khatib R Al-Mafazy AW Ramsan M Rotllant G Gerstenmaier JF Molteni F Abdulla S Montgomery SM Kaneko A Björkman A Impact of artemisinin-based combination therapy and insecticide-treated nets on malaria burden in Zanzibar PLoS Med 2007 4 e309 1784-1790 10.1371/journal.pmed.0040309 17988171 PMC2062481

[B35] Steketee RW Sipilanyambe N Chimumbwa J Banda JJ Mohamed A Miller J Basu S Miti SK Campbell CC National malaria control and scaling up for impact: the Zambia experience through 2006 Am J Trop Med Hyg 2008 79 45 52 18606763

[B36] Chanda P Hamainza B Mulenga S Chalwe V Msiska C Chizema-Kawesha E Early results of integrated malaria control and implications for the management of fever in under-five children at a peripheral health facility: a case study of Chongwe rural health centre in Zambia Malar J 2009 8 49 10.1186/1475-2875-8-49 19292919 PMC2662870

[B37] Chizema-Kawesha E Mukonka V Mwanza M Kaliki C Phiri M Miller J Komatus R Aregawi M Masaninga F Kitikiti S Babaniyi O Otten M Evidence of substantial nationwide reduction of malaria cases and deaths due to scale-up of malaria control activities in Zambia, 2001-2008 World Health Organization, Zambia 19-23 January. Impact Evaluation Mission Report

[B38] Chizema-Kawesha E Miller J Steketee RW Mukonka VM Mukuka C Mohamed A Miti SK Campbell CC The components of success in malaria control: the Zambia Experience Am J Trop Med Hyg 2010 Sep 83 3 480 8 10.4269/ajtmh.2010.10-0035 PMC2929038 20810807

[B39] Kleinschmidt I Sharp B Benavente LE Schwabe C Torrez M Kuklinski J Morris N Raman J Carter J Reduction in infection with *Plasmodium falciparum *one year after the introduction of malaria control interventions on Bioko Island, Equatorial Guinea Am J Trop Med Hyg 2006 74 972 978 16760506

[B40] Kleinschmidt I Torrez M Schwabe C Benavente L Seocharan I Jituboh D Nseng G Sharp B Factors influencing the effectiveness of malaria control in Bioko Island, Equatorial Guinea Am J Trop Med Hyg 2007 76 1027 1032 17556606 PMC3749811

[B41] Kleinschmidt I Schwabe C Benavente L Torrez M Ridl FC Segura JL Ehmer P Nchama GN Marked increase in child survival after four years of intensive malaria control Am J Trop Med Hyg 2009 80 882 888 19478243 PMC3748782

[B42] Roll Back Malaria RBM Progress and Impact Series Reports 2010 1 1 1 95

[B43] Gomez-Elipe A Otero A van Herp M Aguirre-Jaime A Forecasting malaria incidence based on monthly case reports and environmental factors in Karuzi, Burundi, 1997-2003 Malar J 2007 6 129 10.1186/1475-2875-6-129 17892540 PMC2048513

[B44] Protopopoff N Van Bortel W Marcotty T Van Herp M Maes P Baza D D'Alessandro U Coosemans M Spatial targeted vector control is able to reduce malaria prevalence in the highlands of Burundi Am J Trop Med Hyg 2008 79 12 18 18606758

[B45] Mufunda J Nyarango P Usman A Gebremeskel T Mebrahtu G Ogbamariam A Kosia A Ghebrat Y Gebresillosie S Goitom S Araya E Andemichael G Gebremichael A Roll back malaria--an African success story in Eritrea S Afr Med J 2007 97 46 50 17378282

[B46] Nyarango PM Gebremeskel T Mebrahtu G Mufunda J Abdulmumini U Ogbamariam A Kosia A Gebremichael A Gunawardena D Ghebrat Y Okbaldet Y A steep decline of malaria morbidity and mortality trends in Eritrea between 2000 and 2004: the effect of combination of control methods Malar J 2006 5 33 10.1186/1475-2875-5-33 16635265 PMC1501031

[B47] Yeshiwondim AK Gopal S Hailemariam AT Dengela DO Patel HP Spatial analysis of malaria incidence at the village level in areas with unstable transmission in Ethiopia Int J Health Geogr 2009 8 5 10.1186/1476-072X-8-5 19171051 PMC2646707

[B48] Otten M Aregawi M Were W Karema C Medin A Bekele W Jima D Gausi K Komatsu R Korenromp E Low-Beer D Grabowsky M Initial evidence of reduction of malaria cases and deaths in Rwanda and Ethiopia due to rapid scale-up of malaria prevention and treatment Malar J 2009 8 14 10.1186/1475-2875-8-14 19144183 PMC2653503

[B49] Manuel Ramos J Reyes F Tesfamariam A Change in epidemiology of malaria infections in a rural area in Ethiopia J Travel Med 2005 12 155 156 10.2310/7060.2005.12304 15996444

[B50] Graves PM Osgood DE Thomson MC Sereke K Araia A Zerom M Ceccato P Bell M Del Corral J Ghebreselassie S Brantly EP Ghebremeskel T Effectiveness of malaria control during changing climate conditions in Eritrea, 1998-2003 Trop Med Int Health 2008 13 218 228 10.1111/j.1365-3156.2007.01993.x 18304268

[B51] Ceesay SJ Casals-Pascual C Erskine J Anya SE Duah NO Fulford AJ Sesay SS Abubakar I Dunyo S Sey O Palmer A Fofana M Corrah T Bojang KA Whittle HC Greenwood BM Conway DJ Changes in malaria indices between 1999 and 2007 in The Gambia: a retrospective analysis Lancet 2008 372 1545 1554 10.1016/S0140-6736(08)61654-2 18984187 PMC2607025

[B52] Ceesay SJ Casals-Pascual C Nwakanma DC Walther M Gomez-Escobar N Fulford AJC Takem EN Nogaro S Bojang KA Corrah T Jaye MC Taal MA Sonko AJ Conway DJ Continued decline of malaria in The Gambia with implications for elimination PLoS ONE 2010 5 e12242 10.1371/journal.pone.0012242 20805878 PMC2923605

[B53] O'Meara WP Bejon P Mwangi TW Okiro EA Peshu N Snow RW Newton CR Marsh K Effect of a fall in malaria transmission on morbidity and mortality in Kilifi, Kenya Lancet 2008 372 1555 1562 10.1016/S0140-6736(08)61655-4 18984188 PMC2607008

[B54] Okiro EA Hay SI Gikandi PW Sharif SK Noor AM Peshu N Marsh K Snow RW The decline in paediatric malaria admissions on the coast of Kenya Malar J 2007 6 151 10.1186/1475-2875-6-151 18005422 PMC2194691

[B55] Okech BA Mwobobia IK Kamau A Muiruri S Mutiso N Nyambura J Mwatele C Amano T Mwandawiro CS Use of integrated malaria management reduces malaria in Kenya PLoS One 2008 3 e4050 10.1371/journal.pone.0004050 19115000 PMC2603594

[B56] Adazu K Hamel M Feikin D Ofware P Obor D Ogwang S Orimba V Vulule J Slutsker L Laserson K Marked decline in childhood mortality in the Western Kenya DSS: evidence from longitudinal data 2003-2007 Am J Trop Med Hyg 2008 79 Suppl 6 Abstract 372

[B57] Sharp BL Kleinschmidt I Streat E Maharaj R Barnes KI Durrheim DN Ridl FC Morris N Seocharan I Kunene S La Grange JJ Mthembu JD Maartens F Martin CL Barreto A Seven years of regional malaria control collaboration--Mozambique, South Africa, and Swaziland Am J Trop Med Hyg 2007 76 42 47 17255227 PMC3749812

[B58] Guinovart C Bassat Q Sigaúque B Aide P Sacarlal J Nhampossa T Bardají A Nhacolo A Macete E Mandomando I Aponte JJ Menéndez C Alonso PL Malaria in rural Mozambique. Part I: children attending the outpatient clinic Malar J 2008 7 36 10.1186/1475-2875-7-36 18302770 PMC2268704

[B59] Bassat Q Guinovart C Sigauque B Aide P Sacarlal J Nhampossa T Bardají A Nhacolo A Macete E Mandomando I Aponte JJ Menéndez C Alonso PL Malaria in rural Mozambique. Part II: children admitted to hospital Malar J 2008 7 37 10.1186/1475-2875-7-37 18302771 PMC2275288

[B60] Craig MH Kleinschmidt I Le Sueur D Sharp BL Exploring 30 years of malaria case data in KwaZulu-Natal, South Africa: part II. The impact of non-climatic factors Trop Med Int Health 2004 9 1258 1266 10.1111/j.1365-3156.2004.01341.x 15598257

[B61] Craig MH Kleinschmidt I Nawn JB Le Sueur D Sharp BL Exploring 30 years of malaria case data in KwaZulu-Natal, South Africa: part I. The impact of climatic factors Trop Med Int Health 2004 9 1247 1257 10.1111/j.1365-3156.2004.01340.x 15598256

[B62] Barnes KI Durrheim DN Little F Jackson A Mehta U Allen E Dlamini SS Tsoka J Bredenkamp B Mthembu DJ White NJ Effect of artemether-lumefantrine policy and improved vector control on malaria burden in KwaZulu-Natal, South Africa PLoS Med 2005 2 e330 10.1371/journal.pmed.0020330 16187798 PMC1240068

[B63] Gerritsen AA Kruger P van der Loeff MF Grobusch MP Malaria incidence in Limpopo Province, South Africa, 1998-2007 Malar J 2008 7 e162 10.1186/1475-2875-7-162 PMC2538535 18724866

[B64] Sievers AC Lewey J Musafiri P Franke MF Bucyibaruta BJ Stulac SN Rich ML Karema C Daily JP Reduced paediatric hospitalizations for malaria and febrile illness patterns following implementation of community-based malaria control programme in rural Rwanda Malar J 2008 7 167 10.1186/1475-2875-7-167 18752677 PMC2557016

[B65] Teklehaimanot HD Teklehaimanot A Kiszewski A Rampao HS Sachs JD Malaria in Sao Tome and Principe: on the brink of elimination after three years of effective antimalarial measures Am J Trop Med Hyg 2009 80 133 140 19141851

[B66] Tseng LF Chang WC Ferreira MC Wu CH Rampao HS Lien JC Rapid control of malaria by means of indoor residual spraying of alphacypermethrin in the Democratic Republic of Sao Tome and Principe Am J Trop Med Hyg 2008 78 248 250 18256424

[B67] Sarrassat S Senghor P Le Hesran JY Trends in malaria morbidity following the introduction of artesunate plus amodiaquine combination in M'lomp village dispensary, south-western Senegal Malar J 2008 7 215 10.1186/1475-2875-7-215 18950485 PMC2584070

[B68] Kimbi HK Nformi D Patchong AM Ndamukong KJ Influence of urbanisation on asymptomatic malaria in school children in Molyko, South West Cameroon East Afr Med J 2006 83 602 609 17455449 10.4314/eamj.v83i11.9476

[B69] Mabiala-Babela JR Samba-Louaka C Mouko A Senga P Morbidity in a pediatric department (University Hospital of Brazzaville): 12 years later (1989-2001) Arch Pediat 2003 10 650 652 10.1016/S0929-693X(03)00290-2 12907078

[B70] Rabarijaona LP Randrianarivelojosia M Raharimalala LA Ratsimbasoa A Randriamanantena A Randrianasolo L Ranarivelo LA Rakotomanana F Randremanana R Ratovonjato J Rason M-A Duchemin J-B Tall A Robert V Jambou R Ariey F Domarle O Longitudinal survey of malaria morbidity over 10 years in Saharevo (Madagascar): further lessons for strengthening malaria control Malar J 2009 8 190 10.1186/1475-2875-8-190 19660116 PMC3224923

[B71] Himeidan YE Hamid EE Thalib L Elbashir MI Adam I Climatic variables and transmission of falciparum malaria in New Halfa, eastern Sudan East Mediterr Health J 2007 13 17 24 17546901

[B72] Ramroth H Ndugwa RP Muller O Ye Y Sie A Kouyate B Becher H Decreasing childhood mortality and increasing proportion of malaria deaths in rural Burkina Faso Global Health Action 2009 1909 20027271 10.3402/gha.v2i0.1909 PMC2779934

[B73] Orimadegun AE Fawole O Okereke JO Akinbami FO Sodeinde O Increasing burden of childhood severe malaria in a Nigerian tertiary hospital: implication for control J Trop Pediatr 2007 53 185 189 10.1093/tropej/fmm002 17287244

[B74] Weekly malaria surveillance in Zimbabwe Wkly Epidemiol Rec 2003 78 398 400 14651034

